# Noise Reduction for Water Supply Pipeline Leakage Signals Based on the Black-Winged Kite Algorithm

**DOI:** 10.3390/s26020736

**Published:** 2026-01-22

**Authors:** Zhu Jiang, Jiale Li, Haiyan Ning, Xiang Zhang, Yao Yang

**Affiliations:** 1 School of Energy and Power Engineering, Xihua University, Chengdu 610039, China; 0120040037@mail.xhu.edu.cn (J.L.); petrel@mail.xhu.edu.cn (H.N.); zhangxiang@mail.xhu.edu.cn (X.Z.); 0120080007@mail.xhu.edu.cn (Y.Y.); 2 Key Laboratory of Fluid and Power Machinery, Xihua University, Ministry of Education, Chengdu 610039, China

**Keywords:** water supply pipeline, black-winged kite, variational mode decomposition, wavelet thresholding, sample entropy, signal denoising

## Abstract

In order to solve the problem of false alarms and missed alarms in pipeline monitoring caused by a large amount of noise in the negative pressure wave signal collected by pressure sensors, a new pressure signal denoising method based on the black-winged kite algorithm (BWK) is proposed. First, the variational mode decomposition (VMD) parameters are optimized through BWK. Next, the effective modal components are screened by sample entropy, and the secondary noise reduction of the signal is carried out by using the wavelet thresholding (WT). Finally, the signal is reconstructed to achieve noise reduction. Simulation experiments show that, compared with WT and empirical mode decomposition (EMD), the method proposed in this paper can achieve the best noise reduction effect under both high and low signal-to-noise ratio (SNR) conditions. The method proposed in the paper can achieve the highest SNR of 14.2280 dB, compared to WT’s SNR of 12.6458 dB and EMD’s SNR of 5.5292 dB. To further validate the performance of the algorithm, an experimental platform for simulating pipeline leaks is built. Compared with WT and EMD, the method proposed in this paper also shows the best noise reduction effect. This method provides a high-precision and adaptive solution for leak detection in urban water supply pipelines and has strong engineering application value.

## 1. Introduction

**Figure 1:**
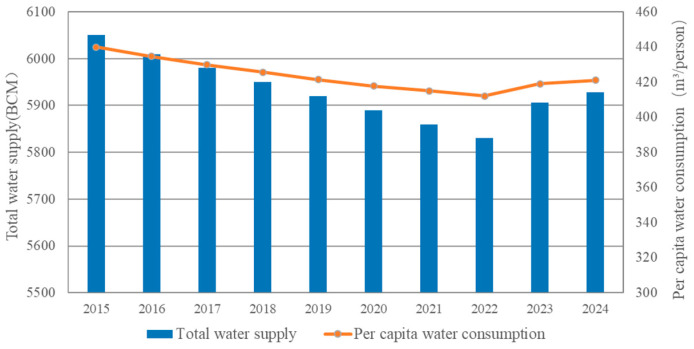
China’s total water supply and per capita water consumption from 2015 to 2024.

Water resources are one of the most precious natural resources on Earth, not only supporting the sustainable development of human society and economy, but also a key factor in maintaining ecosystem stability [1]. According to the “China Water Resources Bulletin” released in 2023, the total volume of water resources in the country reached 2578.25 billion cubic meters, a decrease of 4.82% compared with 2022 [2]. According to the latest data released in the “China Statistical Yearbook” in 2024 [3], from 2015 to 2024, China’s total water supply and per capita water consumption have shown a certain fluctuating trend. The specific data is shown in Figure 1.

With the increasing importance of water resources, the problem of leakage in water supply pipelines has attracted widespread attention from researchers. Many countries have gradually increased their research investment and technological research in this field [4]. Different scholars and researchers have proposed various leakage detection and analysis methods from multiple perspectives. These methods have their own applicability based on different preconditions, and each method has its unique advantages and disadvantages. According to the classification criteria, common classification methods include those (1) based on technical means: hardware detection methods, such as the ground-penetrating radar method [5] and the distributed optical fiber sensing method [6], etc.; software detection methods, such as the acoustic emission detection method [7] and the negative pressure wave method [8], etc.; (2) classified by the objects to be inspected: inspection of the fluid state inside the pipeline [9], the external environment of the pipeline [10], and the condition of the pipe wall [11]; (3) and classified by technical characteristics: traditional methods based on mathematical models and methods based on artificial intelligence technology.

Through a comparative analysis of common detection methods, negative pressure wave (NPW) technology stands out due to its significant advantages of low implementation cost and simple sensor layout. In addition, this technology is suitable for long-distance pipelines and can meet the monitoring needs of large pipeline networks. Its most prominent feature is real-time performance, which can respond promptly to pipeline leaks. With the continuous advancement of artificial intelligence technology and signal processing methods, the application effect of NPW technology in pipeline leakage detection and location has been significantly improved.

On the other hand, the operation of water supply pipelines is also affected by various factors, such as the external environment. For example, external vibrations transmitted through pipelines may interfere with pressure signals. The aging of pipeline materials may also generate additional noise. Interference from power supply lines may also affect pressure sensors and data acquisition devices. These factors may cause the collected pressure signals to contain a large amount of noise. Especially when a pipeline leak occurs, if the NPW signal is disturbed by noise, it will obviously affect the subsequent leak identification and localization. Therefore, this article will focus on the research of noise reduction methods for NPW signals, take effective measures to improve the SNR of signals, ensure the effective extraction of pressure signal features, and provide strong support for subsequent leak detection and leak point localization.

The pipeline pressure signal is a non-predictable signal with non-stationary characteristics. Appropriate methods are needed to suppress the noise in the pipeline pressure signal and improve its signal-to-noise ratio. In recent years, researchers have been dedicated to developing effective algorithms for signal noise suppression and leakage detection methods. Machine learning has achieved remarkable results in this field. Kim et al. [12] proposed a method called LSTM-RNN, which can improve the accuracy and efficiency of leakage source detection. Lei Yan et al. [13] proposed a method combining OPELM and bidirectional LSTM, which can accurately detect leakage events and significantly reduce the number of false alarms compared to existing methods. Liu Ziming et al. [14] proposed a pioneering new neural network architecture, a Kolmogorov–Arnold Network (KAN). To address the limitations of traditional models in handling complex sequence patterns, Remi Gineau et al. [15] proposed the Time Kolmogorov–Arnold Network (TKAN), which makes multi-step time-series prediction more accurate and efficient. However, the existing deep-learning models have undergone a controversial development process, characterized by the continuous expansion of model size, deeper layers, and more network parameters. Nowadays, training a model typically requires hundreds or even thousands of GPU days of computing time, which significantly increases the cost of using artificial intelligence and reduces its ability to be widely applied in daily life [16].

In addition, methods applied in the signal noise reduction domain of leakage detection include those based on the Fourier Transform (FT) [17], filtering techniques [18], wavelet transform (WT) [19], empirical mode decomposition (EMD) [20], and variational mode decomposition (VMD) [21], among others. The basic idea of FT is to convert signals from the time domain to the frequency domain through algorithms and then analyze and process the signals in the frequency domain. Because FT mainly operates in the pure frequency domain, it has high computational efficiency and processing speed. However, the applicable condition of this method is that the signal must be stationary, and the denoising effect on non-stationary signals is relatively weak. A filtering algorithm is a digital signal-processing technique that aims to remove or reduce unwanted components (such as noise, high-frequency interference, etc.) from the original signal through specific mathematical models and methods and extract or enhance useful information. The disadvantages of filtering methods mainly focus on response speed, computational resource consumption, adaptability to rapidly changing signals, and model dependence. When choosing, it is necessary to weigh signal characteristics, real-time requirements, and system resources. The noise reduction method based on WT utilizes the characteristic differences of signals and noise at different wavelet decomposition scales for noise reduction processing. However, at the discontinuous points of the signal, the pseudo-Gibbs phenomenon will occur after wavelet denoising, and the selection of the wavelet threshold has a significant impact on the denoising effect [22]. EMD can adaptively decompose non-stationary signals into a series of Intrinsic Mode Functions (IMFs), each corresponding to a different frequency component of the signal. By removing high-frequency IMFs, the parts dominated by high-frequency noise can be eliminated, thereby reducing the interference of noise on the signal. However, EMD suffers from modal aliasing, which may result in the IMFs obtained after decomposition containing multiple components with different characteristics, or the same characteristics being dispersed into multiple components, seriously affecting the effectiveness of signal denoising [23]. In 2014, Konstantin [24] proposed VMD, which can accurately determine the optimal center frequency and bandwidth of each characteristic mode function by constructing and solving constrained variational problems, effectively alleviating the mode-mixing and endpoint effects that exist in methods such as EMD. However, the decomposition effect of VMD largely depends on its parameter settings, especially the selection of IMFs *K* and penalty factors α. Inappropriate parameter configuration may lead to under-decomposition or over-decomposition of the signal, resulting in signal aliasing or false components. LI et al. proposed a PDS-VMD based on VMD [25], determining *K* by the central frequencies of adjacent IMF under a fixed α. LIU et al. proposed AD-VMD to handle pipeline pressure signals [26], but this method still optimizes the number of IMFs under a fixed penalty factor.

Different signals may have different spectral characteristics and time-domain variations. Simple parameter settings often cannot adapt to all scenarios. Therefore, the method of selecting fixed parameters has significant limitations in practical applications, especially when dealing with diverse or unknown signals. It cannot automatically adapt to the dynamic changes of the signals, thereby affecting the accuracy and effectiveness of the decomposition results. Select the optimal parameter combination $\left[K,\alpha \right]$ in VMD, considering that the BWK optimization algorithm, a new type of meta-heuristic intelligent optimization algorithm, has the advantages of fast search speed, being able to find better solutions in a short period of time, and effectively handling complex optimization problems. Based on the BWK, a novel method named IBWK-VMD-WT for denoising pressure signals is proposed in this paper. First, optimize the number of *K* and the penalty factor α in VMD based on the improved BWK (IBWK) algorithm. Then, effective modal components are screened through sample entropy. Meanwhile, in order to further remove high-frequency noise components from the denoised signal, WT is used for secondary denoising of the signal. Finally, the signal is reconstructed to achieve signal denoising. This article verifies the effectiveness of IBWK-VMD-WT by constructing simulated signals and collecting pipeline leakage signals. The experimental results show that the method proposed in this paper can achieve the best denoising effect on the above two signals.

The rest of this paper is structured as follows. The second part introduces the basic principle of VMD and analyzes the impact of the number *K* of IMFs and the penalty factor α on algorithm performance. It also introduces the proposed method, named IBWK-VMD-WT, in this paper. The experiment will be conducted in the third part. Finally, the conclusions are given in Section 4.

## 2. Methodology

### 2.1. Principle of VMD

VMD is an effective non-stationary signal-processing technique that achieves adaptive decomposition of signals by establishing optimization models.

#### 2.1.1. Optimization Objective

The purpose of VMD is to decompose the input signal $f\left(t\right)$ into *K* modal functions, each of which is a narrowband signal with a definite center frequency. Narrowband signals refer to those whose spectral energy is concentrated within a small range. To ensure that the modal function has narrowband characteristics, VMD generates an analytical signal by applying the Hilbert transformation to the modal function to characterize the frequency band distribution of the modal function. Subsequently, the frequency components of the mode are aligned to the zero-frequency point through spectral translation (i.e., removing the center frequency), thereby accurately measuring and minimizing the bandwidth. Each modal component IMF is represented by Equation (1). To ensure that the bandwidth of all modal functions of the objective function is as narrow as possible, thereby decomposing clear modes, the sum of their bandwidths needs to be taken as the optimization objective, as shown in Equation (2).

(1) $$u_{k}\left(t\right)=A_{k}\left(t\right)\cos\left(\phi_{k}\left(t\right)\right)$$

where $u_{k}\left(t\right)$ is the *k*-th IMF $\left(1\leq k\leq K\right)$. A*_k_* is the instantaneous amplitude of the IMF; $\phi_{k}\left(t\right)$ is the instantaneous phase angle of $u_{k}\left(t\right)$.

(2) $$\underset{u_{k},\omega_{k}}{\min}\sum_{k=1}^{K}\|\partial_{t}\left[\left(\delta \left(t\right)+\frac{j}{\pi t}\right)\cdot u_{k}\left(t\right)\cdot e^{-j\omega_{k}t}\right]{\|}_{2}^{2}$$

where $\delta \left(t\right)+\frac{j}{\pi t}$ is the core of the Hilbert transform, used to construct the analytical signal. $\partial_{t}$ is the derivative of time. $e^{-j\omega_{k}t}$ shifts the signal frequency so that its center frequency $\omega_{k}\left(t\right)$ shifts to the zero-frequency point.

#### 2.1.2. Constraints

To ensure that the original signal does not lose any information due to decomposition, it is necessary to satisfy that the linear superposition of all modal functions must equal the input signal *f*(*t*). That is, Equation (3).

(3) $$f\left(t\right)=\sum_{k=1}^{K}u_{k}\left(t\right)$$

#### 2.1.3. Lagrange Function

To simultaneously optimize the bandwidth of the modal function and meet the constraints of signal reconstruction, VMD introduces Lagrange multipliers and a penalty factor to construct an optimization framework. The augmented Lagrange expression formula is as follows.

(4) $$\Gamma \left(u_{k},\omega_{k},\lambda \right)=\alpha \sum_{k=1}^{K}\|\partial_{t}\left[\left(\delta \left(t\right)+\frac{j}{\pi t}\right)\cdot u_{k}\left(t\right)\cdot e^{-j\omega_{k}t}\right]{\|}_{2}^{2}+\;\|f\left(t\right)-\sum_{k=1}^{K}u_{k}\left(t\right){\|}_{2}^{2}+\langle \lambda \left(t\right),f\left(t\right)-\sum_{k=1}^{K}u_{k}\left(t\right)\rangle$$

The penalty factor α ensures the accuracy of signal reconstruction, while the Lagrange multiplier λ ensures the strictness of constraints.

#### 2.1.4. Optimize the Solution Process

The alternate direction method of the multiplication operator is adopted to alternately update $u_{k}^{n+1}$, $\omega_{k}^{n+1}$, and $\lambda^{n+1}$ to determine the saddle point of the unconstrained variational problem of Formula (4). The formula for updating the variables repeatedly each time is:

(5) $${\hat{u}}_{k}^{n+1}\left(\omega \right)=\frac{\hat{f}\left(\omega \right)-\sum_{i\neq k}{\hat{u}}_{i}\left(\omega \right)+\frac{\hat{\lambda }\left(\omega \right)}{2}}{1+2\alpha {\left(\omega -\omega_{k}\right)}^{2}}$$

Similarly, the updated expression of the center frequency $\omega_{k}$ is:

(6) $$\omega_{k}^{n+1}=\frac{\int_{0}^{\infty }\omega {\left|{\hat{u}}_{k}^{n+1}\left(\omega \right)\right|}^{2}d\omega }{\int_{0}^{\infty }{\left|{\hat{u}}_{k}^{n+1}\left(\omega \right)\right|}^{2}d\omega }$$

Update the Lagrange multiplier using Formula (7):

(7) $${\hat{\lambda }}^{n+1}\left(\omega \right)={\hat{\lambda }}^{n}\left(\omega \right)+\tau \left(\hat{f}\left(\omega \right)-\sum_{k=1}^{K}{\hat{u}}_{k}^{n+1}\left(\omega \right)\right)$$

where ${\hat{u}}_{k}^{n+1}\left(\omega \right)$ is the residual of $\hat{f}\left(\omega \right)-\sum_{i\neq k}{\hat{u}}_{i}\left(\omega \right)$ after Wiener filtering.

Based on the above analysis, the iterative steps of the VMD method are as follows:

Step1: Set the initial values of ${\hat{u}}_{k}^{1}$, $\omega_{k}^{1}$, ${\hat{\lambda }}^{1}$ and *K*;

Step2: Loop iteration: n=n+1; iterate over ${\hat{u}}_{k}$ and $\omega_{k}$ in a loop according to Formulas (5) and (6);

Step3: Iterate through Formula (7) to obtain $\hat{\lambda }$.

Repeat Step1 to Step3 until the condition of (8) is met to stop the iteration.

(8) $$\sum_{k}\|{\hat{u}}_{k}^{n+1}-{\hat{u}}_{k}^{n}{\|}_{2}^{2}/\|{\hat{u}}_{n}^{k}{\|}_{2}^{2}<\varepsilon$$

where ε is the given solution accuracy.

When decomposing the pressure signal inside the pipeline using VMD, it is necessary to set the parameters for decomposition in advance: the number of modes *K*, the penalty factor α, the noise tolerance *tau*, the initialization parameter *init*, whether to remove the *DC* component, and the convergence tolerance (termination condition ε) *tol*. According to the relevant research, the number of modes *K* and α are two main parameters that have a significant impact on the decomposition effect and need to be given special consideration. In addition, other parameters have a relatively small impact on the VMD decomposition. Empirical values are taken, namely *tau* = 0, *init* = 1, *DC* = 0, and *tol* = 10^−7^ [27].

### 2.2. The Influence of the Number of K and α on the Decomposition Effect of VMD

As mentioned earlier, pressure signals in water supply pipelines are affected by factors such as flow rate changes, current noise, etc. For the convenience of analysis, this article selects three cosine signals of 5 Hz, 12 Hz, and 144 Hz components as simulation signals for superposition, in order to analyze the influence of the number of *K* and α on the noise reduction effect. This article mainly analyzes two situations, namely the influence of different numbers of IMFs *K* on the decomposition effect when α is a constant, and the influence of using different α on the decomposition effect when *K* is constant. The simulation signal $S\left(t\right)$ constructed in this paper is shown in Equation (9).

(9) $$x_{1}\left(t\right)=8\cos\left(2\pi 5t\right)\;x_{2}\left(t\right)=5\cos\left(2\pi 12t\right)\;x_{3}\left(t\right)=2\cos\left(2\pi 144t\right)\;S\left(t\right)=x_{1}\left(t\right)+x_{2}\left(t\right)+x_{3}\left(t\right)$$

**Figure 2:**
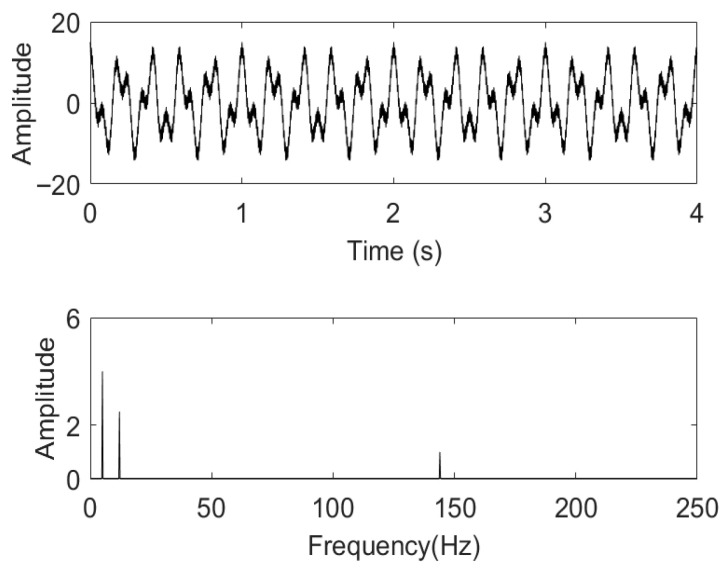
The time-frequency diagram of the simulated signal $S\left(t\right)$.

The time-frequency diagram of the simulation signal $S\left(t\right)$ is shown in Figure 2.

#### 2.2.1. The Influence of the Number *K* of IMFs

**Figure 3:**
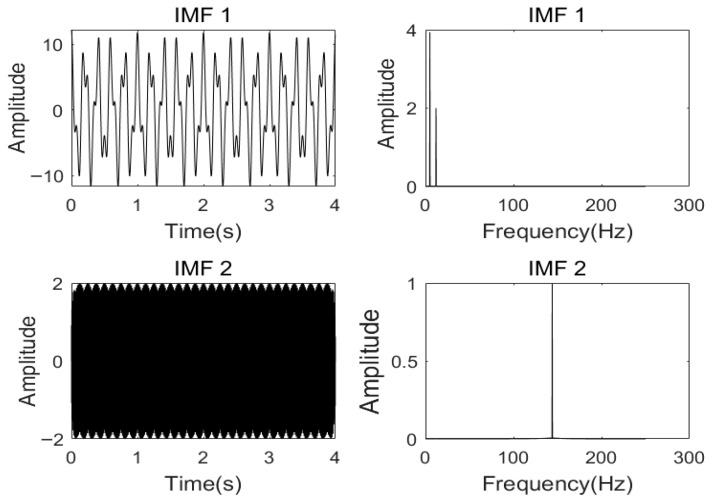
Each mode component and spectrum of VMD decomposition with α=2000, K=2.

**Figure 4:**
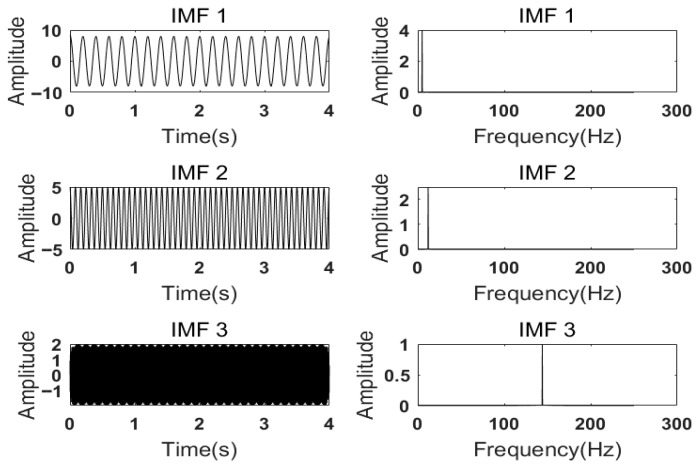
Each mode component and spectrum of VMD decomposition with α=2000, K=3.

**Figure 5:**
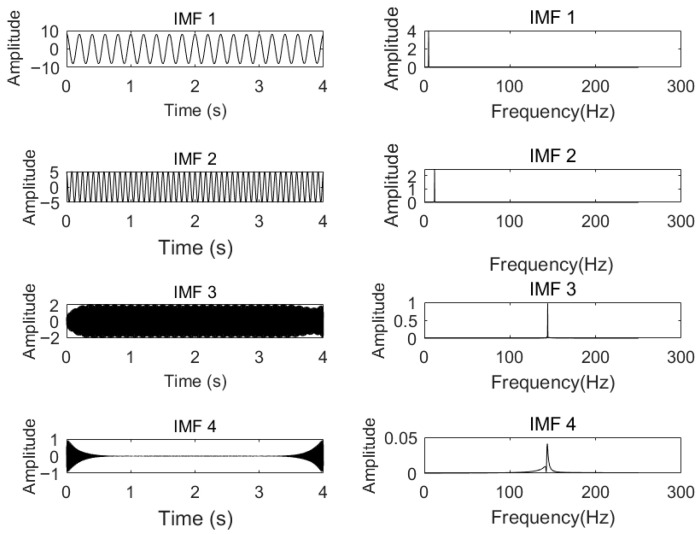
Each mode component and spectrum of VMD decomposition with α=2000, K=4.

When analyzing the influence of the number of *K* of IMFs on the decomposition effect of VMD, it is assumed that the penalty factor α is 2000, and the numbers of *K* are 2, 3, and 4, respectively, to decompose the signal. The corresponding frequency spectra of the modal components obtained are shown in Figure 3, Figure 4 and Figure 5, respectively.

According to Figure 3, when the number of *K =* 2 and α=2000, the simulated signal is decomposed into two components, IMF1 and IMF2. Through the spectrum diagram, it can be seen that the 12 Hz component is not completely decomposed, but is included in other components, resulting in under-decomposition. As shown in Figure 4, when *K* = 3, the three cosine center frequencies of the simulated signal correspond to the center frequencies obtained by decomposing the modal components, and the effect is the most ideal. However, by observing Figure 5, it can be seen that, when *K* = 4 and α=2000, although the frequencies of the first three IMFs almost completely correspond to the simulated signal, the center frequencies of IMF3 and IMF4 obtained by VMD decomposition are the same, resulting in mode aliasing. The simulation experiment results show that, under the condition of α being constant, under-decomposition occurs when the *K* is too small, and mode mixing occurs when the *K* is too large.

#### 2.2.2. The Influence of the Penalty Factor *α*

The following analysis examines the impact of different α on the VMD decomposition performance when *K* is constant.

**Figure 6:**
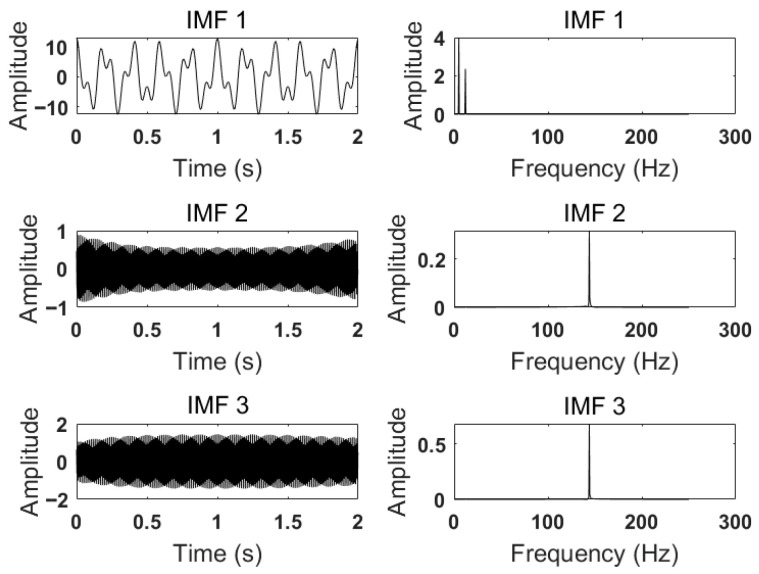
IMFs and spectrum of VMD decomposition with K=3, α=500.

**Figure 7:**
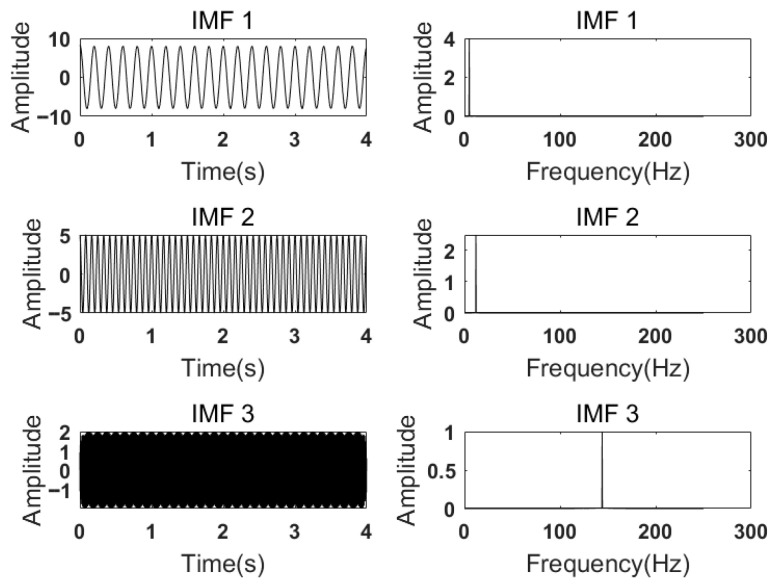
IMFs and spectrum of VMD decomposition with K=3, α=2000.

**Figure 8:**
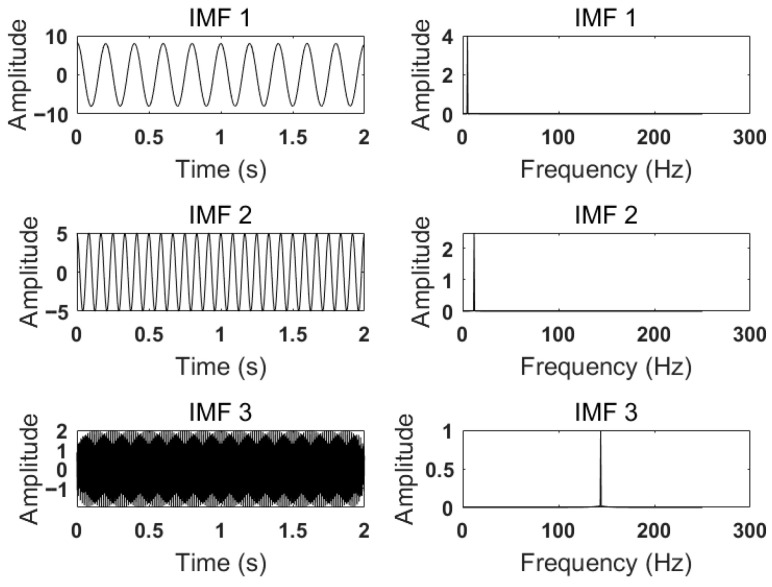
IMFs and spectrum of VMD decomposition with K=3, α=5000.

After setting *K* = 3 and α to 500, 2000, and 5000, respectively, the modal components and corresponding spectra are obtained. The IMFs and corresponding spectra of *K* = 3 and α = 500 are shown in Figure 6. The IMFs and corresponding spectra at *K* = 3 and α = 2000 are shown in Figure 7. The IMFs and corresponding spectra with *K* = 3 and α = 5000 are shown in Figure 8.

According to the results shown in Figure 6, when *K* = 3 and α=500, it can be seen from the comparison of various components of the simulated signal that the 24 Hz signal has not been completely decomposed, resulting in under-decomposition. In addition, the center frequencies of IMF2 and IMF3 are the same, leading to mode mixing. From Figure 7 and Figure 8, it can be seen that the center frequencies of the three IMFs obtained are consistent with the simulated signal, and the VMD decomposition effect is usually ideal. At this time, all IMFs need to be reconstructed to compare and calculate the reconstruction error. Generally, a lower reconstruction error means a better decomposition result. This process checks for excessive smoothing or unnecessary modal aliasing. By calculating when α=2000, the reconstruction error is 0.0025564. When α=5000, the reconstruction error is 0.0041091, indicating that the decomposition effect is optimal when α=2000 and *K* = 3.

However, through the above analysis, it is not difficult to see that determining the number of *K* and α in the VMD based on empirical values often cannot achieve satisfactory results. To solve this problem, this article adopts an optimization algorithm named IBWK-VMD based on improved BWK to optimize *K* and α in order to achieve the best signal decomposition effect and lay the foundation for subsequent signal denoising.

### 2.3. IBWK-VMD

#### 2.3.1. BWK

Heuristic algorithms aim to find the optimal approximate solution to a problem by simplifying its structure and applying empirical rules or exploratory strategies. The focus of the research is mainly on improving the global search capability, local accuracy, and accelerating the convergence process of the algorithm, in order to provide more efficient and accurate search methods for solving complex problems. To further optimize algorithm performance, Wang et al. proposed a novel intelligent optimization algorithm, the BWK, in 2024 [28]. Currently, this algorithm is mainly used to solve problems in engineering disciplines, such as benchmark functions and path planning. For different practical issues, many scholars have proposed improvements to the BWK in order to improve convergence accuracy and stability [29,30,31,32]. The inspiration for this algorithm comes from the hunting and migration behavior of black-winged kites and combines the Cauchy mutation strategy and the Leader strategy, significantly improving global search ability and convergence speed. The core mechanism of BWK includes two key parts: attack behavior and migration behavior.

(1) Initialization phase

The initialization phase is the first step of the algorithm, creating a set of initial solutions in space, such as Formula (10), with the aim of providing a good starting point for subsequent searches, ensuring that individuals can widely cover the entire solution space, and avoiding the algorithm from getting stuck in local optima.

(10) $$X_{i}=BL+rand\left(BU-BL\right)$$

where *rand*() represents taking a random value between 0 and 1. *BL* and *BU* represent the lower and upper bounds of the search range.

(2) Attack behavior

In the BWK algorithm, the simulation of attack behavior is derived from the flight strategy of black-winged kites towards small mammals and insects during hunting. This attack strategy can be divided into two major behavioral patterns: global exploration and local search. Global exploration is manifested as the black-winged kite maintaining balance by extending its wings while hovering in the air, and patrolling its prey over a wide range. This stage reflects the process of the algorithm conducting extensive searches in the solution space, ensuring that the algorithm can cover multiple regions and avoid getting stuck in local optima. And local search simulates the rapid diving and precise approach of a black-winged kite to its prey, representing the algorithm’s focus on a specific local area in the solution space for further refined solution optimization. The following is a mathematical model of the attack behavior:

(11) $$Y_{t+1}^{i,j}=\left\{\begin{array}{c} Y_{t}^{i,j}+n\left(1+\sin\left(r\right)\right)\times Y_{t}^{i,j},p<r\\ Y_{t}^{i,j}+n\times \left(2r-1\right)\times Y_{t}^{i,j},\mathrm{else} \end{array}\right.$$

where $Y_{t}^{i,j}$ and $Y_{t+1}^{i,j}$ represent the position of the *i*-th black-winged kite in the *j*-th dimension during the *t*-th and (*t* + 1)-th iterations, respectively. *r* is the control parameter used to control the nonlinear characteristics of the mapping, with a value range of (0,1). p is a constant.

(3) Migration behavior

To cope with environmental factors such as climate change and food supply, birds often migrate in search of more suitable living conditions and resources. During migration, there is usually a leading bird responsible for guiding the entire group, and its navigation ability is crucial to the success or failure of the migration. The BWK algorithm mimics this natural phenomenon and adopts the leader strategy to enhance search efficiency. Specifically, when the fitness value of a certain population is lower than that of a random population, it indicates that the individual is no longer suitable for leadership and thus will be deprived of leadership and transferred to a migratory population. If the fitness of this individual is superior to that of a random population, it will continue to lead and guide the group in the right direction until it reaches the target position. Through the mechanism of dynamically selecting leaders, BWK can ensure the smooth progress of migration, enhance the global search ability, and thereby improve the optimization effect of the algorithm.

The mathematical model of the migration behavior of BWK:

(12) $$Y_{t+1}^{i,j}=\left\{\begin{array}{c} Y_{t}^{i,j}+C\left(0,1\right)\times \left(Y_{t}^{i,j}-L_{t}^{j}\right),F_{i}<F_{r,i}\\ Y_{t}^{i,j}+C\left(0,1\right)\times \left(L_{t}^{j}-m\times Y_{t}^{i,j}\right),\mathrm{else} \end{array}\right.$$

(13) $$m=2\times \sin\left(r+\pi /2\right)$$

where $L_{t}^{j}$ is the leading scorer of the black-winged kite in the *j*-th dimension of the *t*-th iteration up to now. $F_{i}$ is the current position of any black-winged kite in the *j*-th dimension obtained in the *t*-th iteration. $F_{ri}$ is the fitness value of any black-winged kite at a random position in the *j*-th dimension obtained in the *t*-th iteration. m is the correction factor. $C\left(0,1\right)$ is the Cauchy mutation.

From the parameter optimization process of the BWK, it can be seen that measurement indicators play an important role, and the fitness value is a key tool for evaluating the rationality of parameter combinations. In order to improve the optimization accuracy and effectiveness of the algorithm, envelope entropy is usually used as the objective function to find the parameter combination that minimizes the envelope entropy. In general, the minimum envelope entropy indicates that the modal structure of the signal is the most ordered, with the least noise and redundant components.

Envelope entropy [33] is a measure of the complexity, disorder, or information content of a signal. In signal processing, it reflects the degree of dispersion of the probability distribution of the signal envelope. This process calculates the envelope of the signal using Hilbert transform and then normalizes it. Finally, select the minimum value of the envelope entropy of all modalities as the output of the fitness function. For each modal signal, the formula for calculating envelope entropy is:

(14) $$H\left(x\right)=-\sum_{i=1}^{N}p_{i}\log\left(p_{i}\right)$$

where $p_{i}$ is the probability value of the normalized envelope signal, and *N* is the length of the envelope signal. In Section 2.3.3, we constructed the simulation signals and analyzed and compared the values of the envelope entropy and the convergence speed of the algorithms for each method.

#### 2.3.2. Improved BWK

Although the BWK algorithm performs well in a global search and has a high solution quality in complex solution spaces, there are still some limitations, such as population initialization leading to getting stuck in local optima, slow convergence speed, and low local search accuracy. To overcome these shortcomings, an improved BWK algorithm (IBWK) is proposed in this paper that introduces three improvement strategies: tent chaotic mapping initialization, lens-imaging reverse learning, and golden sine strategy. The combination of these three strategies can not only improve the performance of BWK in complex optimization problems, but also enhance its global search ability, accelerate convergence speed, and improve the accuracy of local search.

(1) Tent chaotic mapping

In the classic BKA algorithm, population initialization is usually conducted randomly, which may lead to uneven distribution of individuals in the initial stage, thereby affecting the efficiency of global search. To address this issue, this paper introduces tent chaotic mapping [34] for population initialization. Tent chaotic mapping generates uniformly distributed individuals through nonlinear mapping relationships, ensuring that the initial population can cover the entire solution space and avoid excessive concentration in certain regions. This improvement effectively enhances the diversity of the algorithm and improves its global search capability. The recursive formula for tent mapping is as follows.

(15) $$x_{i}\left(n+1\right)=\left\{\begin{array}{c} \frac{x_{i}\left(n\right)}{r}\\ \frac{1-x_{i}\left(n\right)}{1-r} \end{array}\right.$$

where $x_{i}\left(n\right)$ represents the value of the *i*-th individual in the *n*-th generation.

(2) Lens-imaging reverse learning

In the BWK, attack behavior is one of the core mechanisms, responsible for exploring different regions in the solution space. Lens-imaging reverse learning [35] is an optimization strategy that simulates the principle of physical lens imaging. Through the refraction and focusing characteristics of light, it guides individuals to precisely adjust their positions to approach the optimal solution. Introducing lens-imaging reverse learning into the design of attack behaviors can effectively improve search accuracy, especially in the local search stage. When the individual position is updated, lens-imaging reverse learning guides the search direction and controls the step size, avoiding excessive position adjustments, thereby ensuring that the individual can precisely approach the optimal solution. During the process of attack behavior, lens-imaging reverse learning controls the individual search step size by adjusting the exponential decay factor and gradually converges to the region where the optimal solution is located. The specific mathematical formula is as follows:

(16) $$XPosNew\left(i,:\right)=\frac{BU-BL}{2}+\frac{BU+BL}{2k}-\frac{XPos\left(i,:\right)}{k}$$

where *k* is the exponential decay factor and $k={\left(1+{\left(t/T\right)}^{0.2}\right)}^{6}$. *t* is the current iteration count, and *T* is the total iteration count. *k* gradually decreases with the increase of iteration times, thus avoiding instability caused by large step sizes and optimizing the local search process.

(3) Golden Sine Strategy

The improved method in the paper, the novel metaheuristic algorithm Golden sine algorithm (GSA) proposed by Tanyildizi et al. [36], is used as the search strategy to enhance the global capability and local accuracy of the search process by adjusting the position of individuals. The golden sine strategy combines the idea of the golden ratio and aims to balance global and local search. By optimizing the step size and adjusting the search direction, it improves the convergence speed and overall performance of the algorithm. The basic principle is to apply the golden ratio rule to design the search step size and achieve periodic adjustment through a sine function. Specifically, first generate two parameters $x_{1}$ and $x_{2}$ related to the golden ratio using a random value, as shown in Formula (16), and then update the individual position through these two parameters. The formula for position update is shown in (17):

(17) $$x_{1}=a+\left(1-\varphi \right)\cdot \left(b-a\right)\;x_{2}=a+\varphi \cdot \left(b-a\right)$$

where a and b represent the left and right endpoints of the current search interval, usually chosen as the upper boundary *BU* and the lower boundary *BL* of the search. φ represents the golden section coefficient with a value of $\left(\sqrt{5}-1\right)/2$.

(18) $$x_{i}^{\mathrm{new}}\left(\nu \nu \right)=x_{i}\left(\nu \nu \right)\cdot |\sin\left(r_{1}\right)|-r_{2}\sin\left(r_{1}\right)\cdot |x_{1}\cdot \mathrm{bestPos}\left(\nu \nu \right)-x_{2}\cdot x_{i}\left(\nu \nu \right)|$$

where $x_{i}^{\mathrm{new}}\left(\upsilon \upsilon \right)$ represents the new position of the *i*-th individual in the νν dimension. $x_{1}$ and $x_{2}$ are two reference points calculated based on the golden section ratio. $\mathrm{bestPos}\left(\nu \nu \right)$ is the value of the global optimal solution in the νν dimension, and $x_{i}\left(\nu \nu \right)$ is the value of the current individual in the νν dimension.

In summary, the operation steps for optimizing the VMD parameters *K* and α (referred to as IWBK-VMD in the paper) using the improved black-winged kite algorithm (IWBK) are as follows:

Step 1: Set the initialization parameters of BWK, determine the minimum envelope entropy as the fitness function, and introduce the tent chaotic mapping to initialize the population;

Step 2: Calculate the fitness value of each individual within the population through traversal;

Step 3: Use Equations (11), (16), (12) and (18) to compare the fitness values during the aggressive behavior and migration behavior. Continuously update the individual positions. If the fitness of the new individual is smaller than that of the original one, replace the position of the previous one with that of the new individual; otherwise, retain the original individual. When the number of iterations reaches the maximum value, stop the iteration and output the minimum global fitness value and the optimal parameter combination $\left[K,\alpha \right]$.

**Figure 9:**
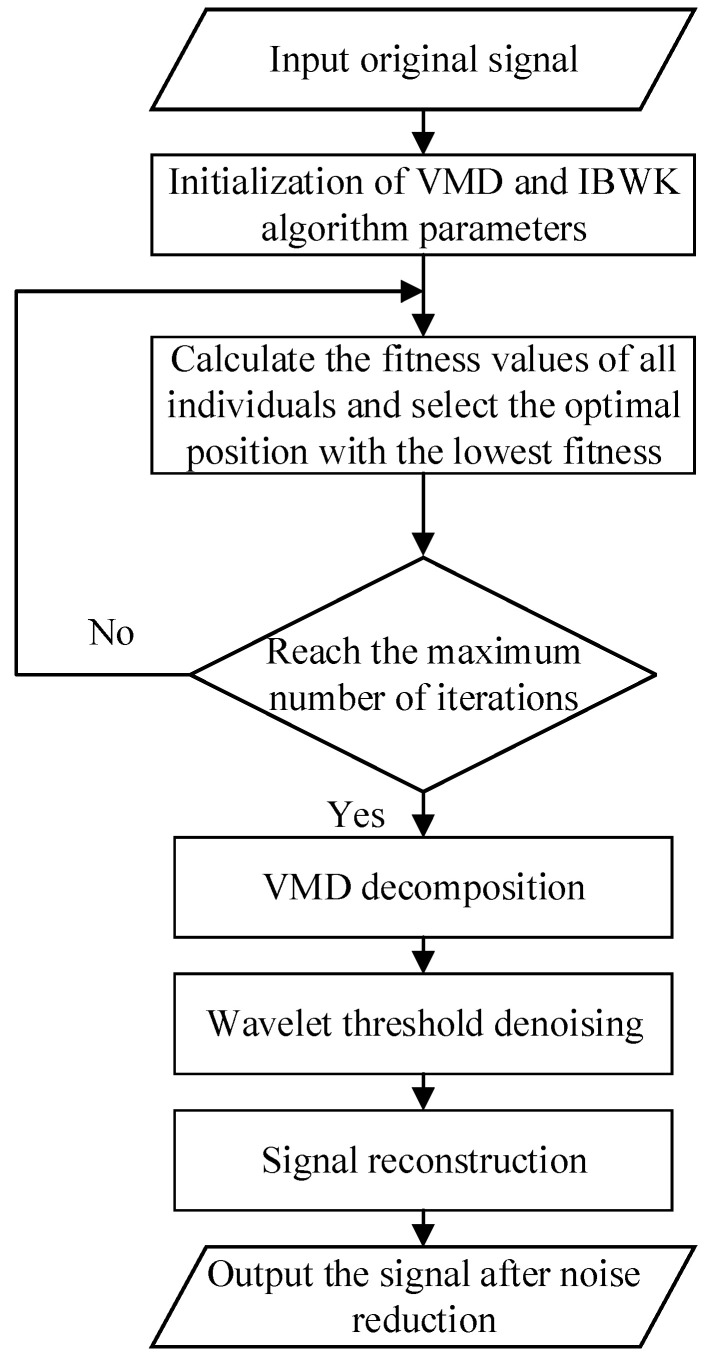
IBWK-VMD-WT noise reduction method flowchart.

The flowchart of the improved algorithm is shown in Figure 9.

#### 2.3.3. Simulation Experiment

To verify the performance of IBWK-VMD, we constructed a simulation signal, as shown in Equation (19). The simulated signal is composed of multiple components, including cosine signals of three different frequencies and Gaussian noise. The frequencies of these three cosine signals are set to 5 Hz, 12 Hz, and 144 Hz, respectively. η is a Gaussian white noise with an SNR of 10 dB. In addition, the sampling frequency of the signal is set to 500 Hz, and the number of sampling points is 3000.

(19) $$X\left(t\right)=8\cos\left(2\pi 5t\right)+5\cos\left(2\pi 12t\right)+2\cos\left(2\pi 144t\right)+\eta$$

To verify the performance advantages of the IBWK-VMD algorithm based on the minimum envelope entropy proposed in this chapter, we compare it with two other optimization algorithms (BKA-VMD [37] and NGO-VMD [38]) of the same type. The number of particles is set to 20, the number of iterations is 20, and the parameter value ranges are respectively set to [2, 10] and [1000, 5000]. In this comparative experiment, all three selected optimization algorithms adopted the fitness function proposed in this chapter. The number is designed, and when optimizing the VMD parameters, the search range of each method remains consistent to ensure the fairness of the comparison results. The optimization results of the three algorithms for *K* and α are shown in Table 1.

According to the results listed in Table 1, the standard deviation of the *K* of the IBWK-VMD algorithm is the smallest, indicating that it performs particularly stably in optimization. The *K* of the BWK-VMD and NGO-VMD algorithms varies greatly, showing significant fluctuations. In contrast, in the optimization of the α, IBWK-VMD also shows strong stability, with the smallest fluctuation range of α, while BWK-VMD and NGO-VMD show relatively large fluctuations in value changes. Overall, the IBWK-VMD demonstrates good stability in the process of signal optimization.

In addition, we also calculated the fitness functions of the three methods. Among them, IBWK-VMD successfully converged at the second iteration, and its minimum fitness value was 5.45313. BKA-VMD and NGO-VMD converge at the fourth iteration, with minimum fitness values of 6.72503 and 6.72379, respectively. As mentioned earlier, fitness value is a key criterion for measuring the quality of optimized solutions. In the process of minimum envelope entropy optimization, a smaller fitness value means that the decomposed modal signal is more ordered and has fewer noise components, resulting in better optimization performance. Therefore, by comparing the convergence speed and fitness values of different optimization algorithms, it can be clearly seen that IBWK-VMD performs well in iteration speed, and its minimum fitness value is also lower than the other two algorithms. This indicates that IBWK-VMD has demonstrated high efficiency and ideal optimization results during the optimization process.

As mentioned earlier, during the operation of water supply pipelines, pressure signals are subject to various types of noise interference. We conducted extensive experiments in the laboratory and found that using the IBWK-VMD algorithm can adaptively adjust the parameters of VMD, enabling the optimized VMD decomposition to better separate signals and noise, thereby reducing the impact of noise and improving the quality of modal decomposition. However, when VMD processes complex signals or signals containing strong noise, the effective components may still contain noise, and directly reconstructing the effective components may reintroduce noise components or irrelevant information into the final signal, resulting in signal distortion or distortion.

To address this issue, this paper proposes a novel method named IBWK-VMD-WT denoising based on WT. The effective components obtained from IBWK-VMD decomposition are subjected to secondary denoising using wavelet thresholding, and the IMF components after secondary denoising are reconstructed to obtain the denoised signal.

### 2.4. IWBK-VMD-WT Denoising

#### 2.4.1. Wavelet Thresholding

The core idea of the wavelet transform is to reveal the multi-level characteristics of the signal by decomposing the original signal into different frequency components. During this process, the low-frequency part usually contains the main information of the signal, while the high-frequency part mostly contains the high-frequency noise of the signal. By processing these frequency components differently, the wavelet transform can effectively capture local information in the signal, especially the detailed changes in the signal mutation region. It is precisely for this reason that the wavelet transform is widely applied in fields such as signal processing and image denoising [21].

In wavelet denoising, a common method is WT. This method achieves the effect of denoising by setting appropriate threshold functions to process the low-frequency and high-frequency parts obtained after the wavelet transform, respectively. Specifically, after the signal undergoes wavelet transform, it is decomposed into low-frequency and high-frequency components. The wavelet coefficients in the low-frequency part are relatively large and can capture the main characteristics of the signal, which are usually retained. The wavelet coefficients in the high-frequency part are relatively small and usually contain more noise components, so these coefficients will be removed. By setting the appropriate threshold, noise can be effectively removed while retaining the main information of the signal. The core steps of wavelet threshold noise reduction include:

(1) Select the appropriate wavelet basis function and the number of layers of wavelet decomposition;
(2) Select an appropriate wavelet threshold function and set the threshold;
(3) Perform the inverse wavelet transform and reconstruct the original signal through threshold processing of the wavelet coefficients.

The effect of wavelet noise reduction is influenced by multiple factors, including the selected wavelet basis function, the number of decomposition layers, the threshold selection, etc. When applying the wavelet threshold method to process noise signals, the primary task is to select the appropriate wavelet basis. Common orthogonal wavelet basis functions include Haar, dbN, Coiflet, Sym, etc. This paper will mainly adopt the Sym wavelet function for signal-denoising processing, as it demonstrates a good balance between smoothness and computational efficiency.

After choosing the appropriate wavelet basis, the next step is to determine the suitable number of wavelet decomposition layers. An excessively high number of decomposition layers may lead to the excessive decomposition of low-frequency information, thereby losing valuable signal information and increasing the computational burden at the same time. However, if the number of decomposition layers is too low, it may not be able to effectively decompose the noise signal, resulting in the inability to remove the excessive high-frequency noise. Therefore, a reasonable number of decomposition layers is crucial to the noise reduction effect. After experimentation, this article has determined that the number of decomposition layers is 6.

After completing the wavelet decomposition, choosing the appropriate threshold and threshold function is also the key to ensuring the noise reduction effect. Common threshold functions include hard threshold functions and soft threshold functions. Each method has different effects in denoising, and a choice can be made based on different actual needs.

The formula for the hard threshold function is as follows:

(20) $$y_{i}=\left\{\begin{array}{c} x_{i},\mathrm{if}{|x}_{i}|>T\\ 0,\mathrm{if}{|x}_{i}|\leq T \end{array}\right.$$

The formula for the soft threshold function is as follows:

(21) $$y_{i}=sign\left(x_{i}\right)\max\left(|x_{i}|-T,0\right)$$

where $y_{i}$ represents the wavelet coefficients after soft and hard threshold processing, $x_{i}$ is the wavelet coefficient of the original signal, and *T* is the threshold. Compared with the soft threshold function, the hard threshold function is smoother, avoiding possible sudden changes in the signal and better preserving the continuity of the signal during denoising. Therefore, this paper opts to adopt the soft threshold function.

#### 2.4.2. Objective Function

Sample entropy (SpEn) [38] is an important tool for evaluating the complexity of time series, and it is used to quantify the degree of irregularity and dynamic changes in signals. Unlike traditional entropy measures (such as Shannon entropy), sample entropy is more suitable for analyzing nonlinear and non-stationary signals and can effectively distinguish the ordered and disordered parts of the signals. Since VMD decomposition may introduce noise components, these noise components usually exhibit higher sample entropy, while the modes representing the effective signals show lower sample entropy values. Therefore, by screening based on sample entropy, it is possible to effectively distinguish between noise and signals, thereby identifying the most representative effective modes. Specifically, sample entropy reflects the changes in signal patterns by measuring the complexity of similar patterns in a time series. In the calculation, sample entropy takes into account the similarity of adjacent data points. If the data variation pattern is relatively simple or consistent, the sample entropy value is relatively low. If the data fluctuates greatly or shows a high degree of irregularity, the sample entropy value is relatively high. When the original signal is decomposed into K IMFs through VMD, the fewer noise components contained in the IMFs, the stronger the correlation with the original signal, and the smaller the SpEn and vice versa. In this article, considering that sample entropy calculation is simple and does not require complex model assumptions, it can quickly process large-scale data, avoid redundant information in the data, and thus improve the accuracy of its estimation. In addition, compared to traditional entropy calculation methods, sample entropy has strong robustness to noise in the data. Therefore, this article takes minimizing sample entropy as the objective function.

The mathematical expression for sample entropy is

(22) $$SpEn=-\ln\left(\frac{C\left(r,m,N\right)}{C\left(r,m+1,N\right)}\right)$$

where $C\left(r,m,N\right)$ represents the matching probability, r is the tolerance, *m* is the embedding dimension of the time series, and N is the number of data points.

However, VMD cannot completely eliminate all noise components when facing noise, especially when dealing with complex signals or signals containing strong noise. The effective components may still contain noise, and directly reconstructing the effective components may reintroduce noise components or irrelevant information into the final signal, resulting in signal distortion or distortion. To solve this problem, this paper uses wavelet thresholding denoising to perform secondary denoising on the effective components obtained from VMD decomposition and reconstructs the IMF components after secondary denoising to obtain the denoised signal.

#### 2.4.3. IBWK-VMD-WT Noise Reduction

The specific noise reduction process of method IBWK-VMD-WT proposed in this article is as follows.

First, use the three steps introduced in Section 2.3.2 to optimize the VMD parameter combination and obtain the optimal $\left[K,\alpha \right]$.

Second, to further remove high-frequency noise from the effective components, the WT is used to perform secondary denoising on the effective IMF components.

Finally, reconstruct the secondary denoising component to obtain a denoised signal.

## 3. Experimental Analysis

To verify the effectiveness of the noise reduction method proposed in this article, we will analyze the noise reduction effect using two types of signals: simulated signals and NPW signals from the leakage simulation test platform.

### 3.1. Simulation Signal Denoising

The simulated signal is shown in Equation (23), and the sampling frequency is 500 Hz The number of sampling points is 3000.

(23) $$F=\sin\left(360\pi \right)+\sin\left(120\pi +0.6\right)+\sin\left(6\pi +1\right)$$

**Figure 10:**
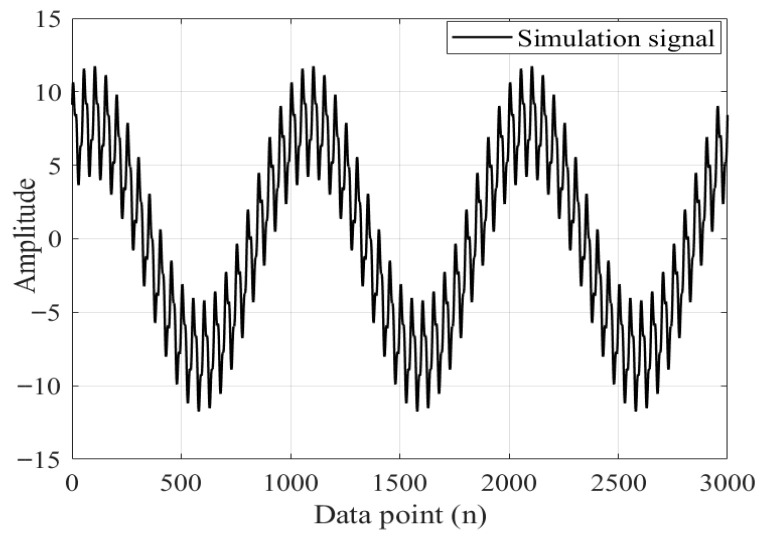
Simulation signal F.

After adding Gaussian white noise with an SNR of 4 dB to the simulated signal F, the simulated signal is shown in Figure 10.

To ensure the comparability of the results of different methods, the above noise reduction results can be quantified by the value of SNR (see Equation (24)) to avoid subjective judgment.

(24) $$SNR=10\mathrm{lg}\left(\frac{P_{s}}{P_{n}}\right)$$

where $P_{s}$ is the power of the signal, and $P_{n}$ is the power of the noise signal.

**Figure 11:**
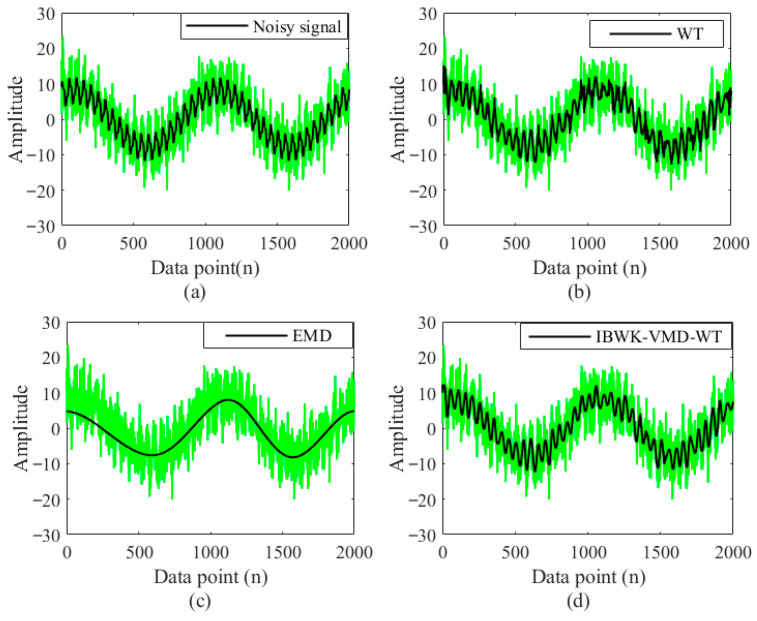
Comparison plots of noise reduction signals: (**a**) noisy signal; (**b**) WT; (**c**) EMD; (**d**) IBWK-VMD-WT.

To verify the superiority of the denoising method proposed in this paper, WT [39] and EMD [34] were used, and the proposed method was applied to denoise the noisy signal shown in Figure 11. In order to highlight the key features of the signal, only 2000 data points of the noise signal are shown in the figure.

As shown in Figure 11a, the black lines represent the original signal without noise, while the green lines represent the signal containing noise. It can be observed from the figure that the noisy signal (the green line) still maintains a certain overall trend, and its contour is similar to the original signal. However, due to the addition of noise, the high-frequency components in the signal increase, causing the high-frequency details to be masked by the noise and reducing the clarity and accuracy of the signal. Figure 11b, c, and d, respectively, present the results of processing noisy signals by three different noise reduction methods. The green curve represents the simulated signal with noise, while the black line represents the signal after denoising using different methods.

The WT selects the basis function “Sym” and decomposes it into six layers. The SNR of the denoised signal is 12.6458 dB. As shown in Figure 11b, the denoised signal still contains a large amount of noise. According to Figure 11c, it can be seen that there is severe distortion in the waveform after EMD denoising, with an SNR of 5.5292 dB. However, the denoising effect of the method proposed in this paper is shown in Figure 11d. The denoised signal is generally good and highly consistent with the simulated signal without noise, with an SNR of 14.2280 dB. The specific comparison is shown in Table 2. According to the results in Table 2, the method proposed in this paper has the best denoising effect.

**Figure 12:**
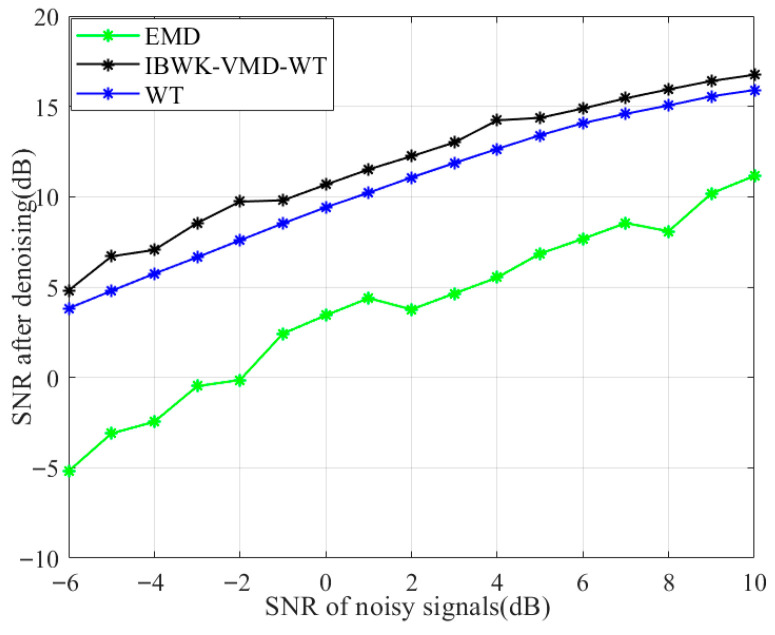
SNR corresponding to different methods.

In order to further verify the denoising effect of the proposed method in this article, the above-mentioned methods were used to denoise signals with different signal-to-noise ratios. The results are shown in Figure 12, where the horizontal axis represents the signal-to-noise ratio of the noise signal before denoising, and the vertical axis represents the signal-to-noise ratio of the noise signal after denoising.

As the SNR gradually increases, the denoising effect of all methods has improved. The performance of EMD is relatively poor under low SNR, especially with its ability to suppress noise being weak. Combining with Figure 11c, it can be seen that there is a distortion phenomenon. The performance of WT is relatively stable, although its denoising effect has limited improvement when the SNR is low. The effect also improves significantly with the increase of SNR. In contrast, the IBWK-VMD-WT exhibits excellent denoising performance in various SNR ranges and, overall, outperforms WT. In summary, the noise reduction effect of our method is relatively stable and better than others, making it more suitable for noise reduction of pipeline leakage signals under pressure sensors.

### 3.2. NPW Signal Denoising

During the stable operation of urban water supply pipelines, objective factors such as fatigue, temperature changes, chemical erosion, lack of maintenance, and natural disasters can lead to leakage. Near the leakage point, a sudden drop in liquid flow velocity creates a large negative pressure area. Liquid molecules in this area transfer energy through collisions and vibrations, forming a wavefront area of NPW, which is characterized by a sharp drop in pressure. If a pressure sensor is installed at the pressure pipe of the pipeline at a certain distance from the leakage point, it will capture the negative pressure wave signal. The accurate extraction of this signal feature lies at the foundation of fault detection and localization. After verifying the performance of the algorithm with simulation signals, we further confirmed the effectiveness of the algorithm by building a leakage simulation test platform.

**Figure 13:**
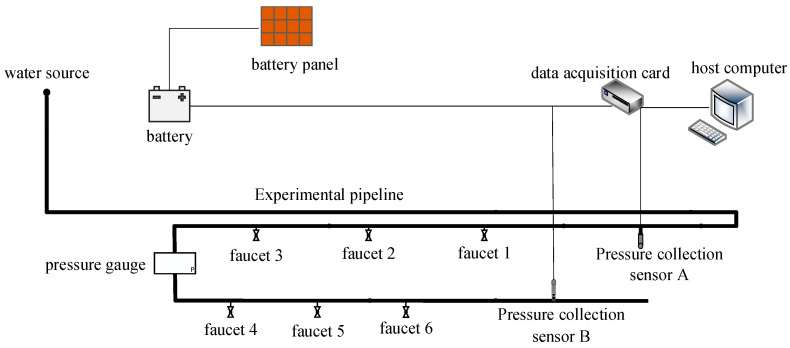
Topology diagram of leakage simulation test platform.

**Figure 14:**
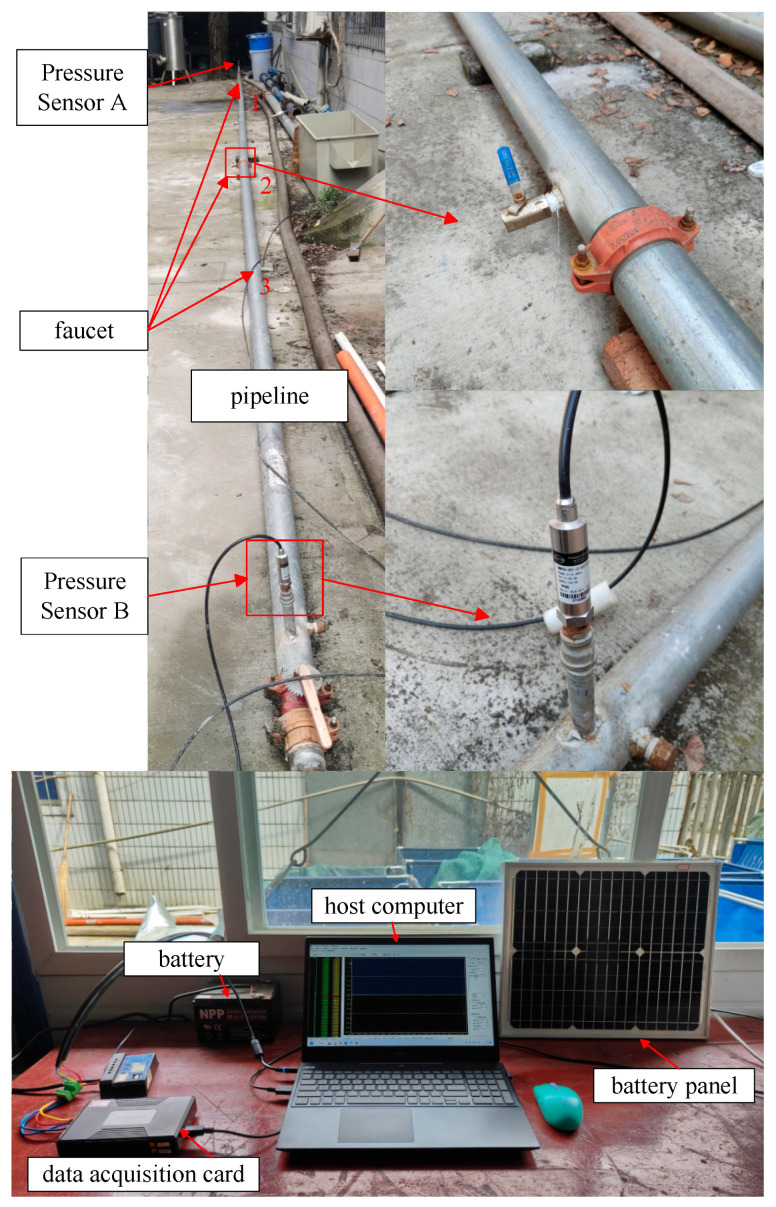
Experimental platform.

The topology diagram of the water supply pipeline system constructed in this article is shown in Figure 13. Partial experimental equipment and data acquisition equipment are shown in Figure 14. The experimental pipeline is supplied with water through the tap-water pipe connected to the fire hydrant, so that the pressure signal characteristics generated during the experiment are similar to those in the actual urban water supply pipeline. The installation of multiple faucets is used to simulate pipeline leaks and simulate leakage situations at different locations. Pressure sensors A and B are installed on the pipeline to collect real-time pressure data inside the pipeline. Solar panels are used to supplement electrical energy for batteries, which provide stable power to sensors. The data acquisition card is responsible for uploading the signal data collected by the sensors to the upper computer, with a sampling frequency set at 500 Hz to meet the real-time requirements of data transmission.

**Figure 15:**
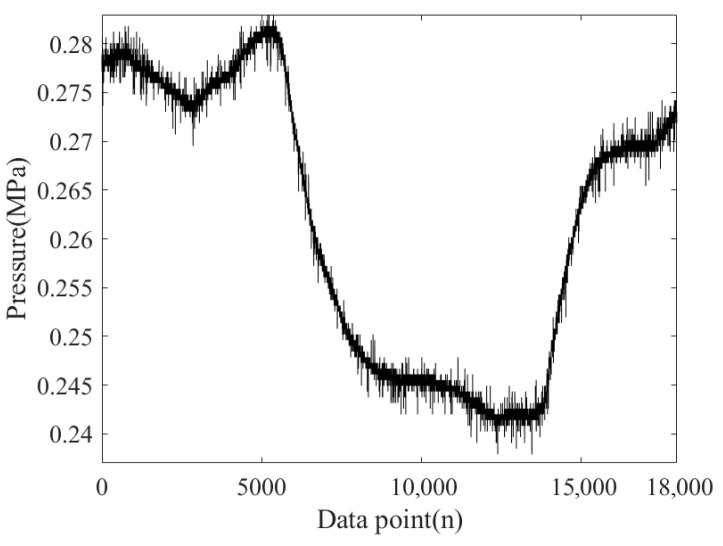
NPW signal.

Figure 15 shows the NPW signal collected by sensor A during laboratory pipeline leakage under experimental conditions, with a total of 18,000 data points collected. From Figure 15, it can be seen that there is significant noise in the signal, especially random fluctuations between the data points and the sharp noise peaks. These noises may be caused by factors such as interference from the experimental environment or pipeline leaks. If these noises are not effectively removed, they will affect subsequent signal processing and feature extraction. Therefore, choosing the appropriate denoising method is crucial.

**Figure 16:**
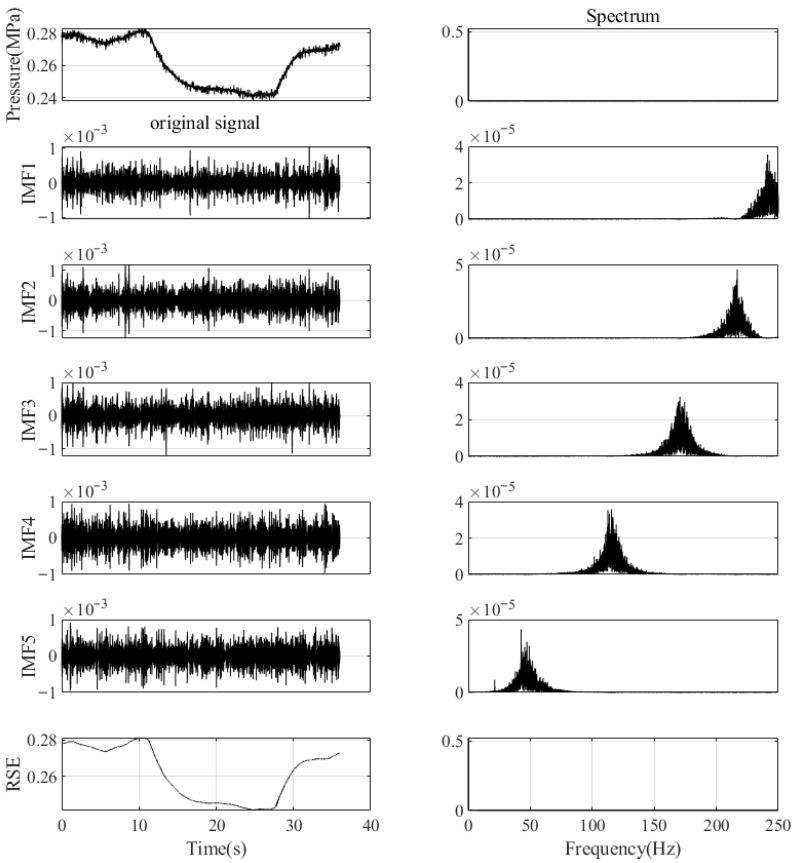
Each IMFs and its corresponding spectral diagram.

By using the IBWK-VMD, the optimal VMD parameters *K =* 6 and α = 3036 were obtained. Next, the NPW signal is decomposed into multiple IMFs based on the parameters, and the frequency diagram of each IMF corresponding to the signal is shown in Figure 16.

According to the frequency distribution of different IMF components shown in the spectrum, it can help distinguish noise from signals. The frequency gradually transitions from high to low, and there is no proximity or overlap in the frequency center, indicating that there is no frequency aliasing between the modes. At the same time, the reconstruction error (RSE) curve in the figure also shows the degree of signal restoration. A lower RSE value indicates that VMD performs well in decomposing and reconstructing signals, and the denoising effect is significant.

**Figure 17:**
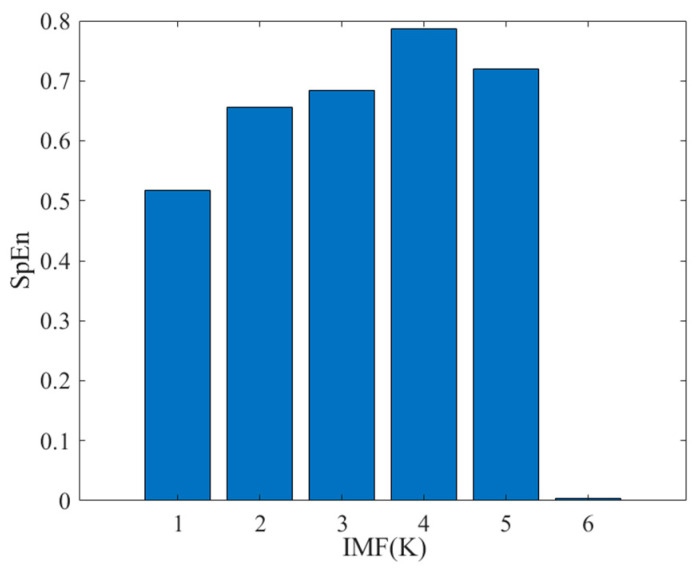
Sample entropy of each IMF.

Then, the sample entropy of each decomposed IMF is calculated, and the above IMF is divided into noise IMF components and effective IMF components based on the sample entropy. Table 3 and Figure 16 present the values of sample entropy for each variational mode function. By analyzing the SpEn in Table 3 and Figure 17, it can be found that the sample entropy values of IMF1 to IMF5 are relatively high, indicating that these modal components contain a lot of effective information. In contrast, the sample entropy of IMF6 is 0.0026, much lower than that of the other IMF components, which means that it may be a noise component and can be removed. The next steps will be to perform wavelet thresholding denoising on IMF1 to IMF5, removing noise and preserving useful parts of the signal, thereby improving signal quality. Through comprehensive analysis of the charts, effective IMFs were screened using the sample entropy method, significantly improving signal quality, removing noise, and preserving the essential characteristics of the signal. Furthermore, it has been verified that sample entropy, as a tool for screening signals, has important rationality and effectiveness in signal noise reduction.

**Figure 18:**
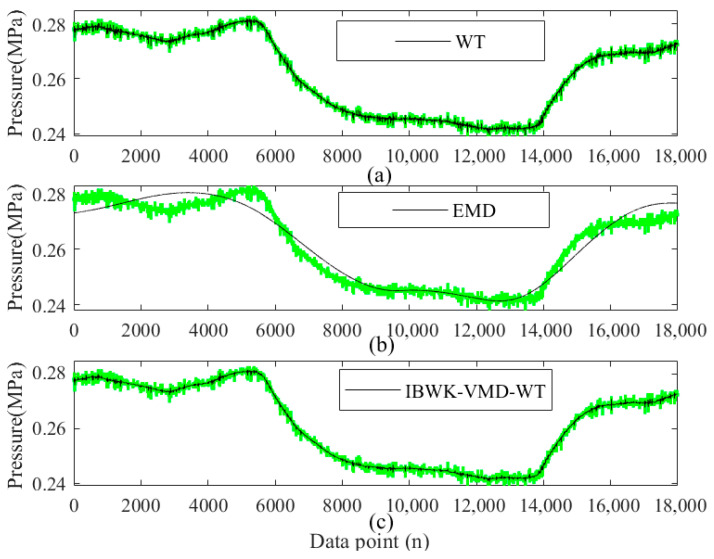
Noise reduction effect of different methods for signal in leaky state: (**a**) WT; (**b**) EMD; (**c**) IBWK-VMD-WT.

Finally, based on the sample entropy screening, secondary denoising is performed using WT. Simultaneously introducing WT and EMD to compare signal denoising, the results are shown in Figure 18. The green line in the figure represents the original negative pressure wave signal before noise reduction, and the black line represents the result after noise reduction. Figure 18a shows that the WT effectively removes high-frequency noise while preserving the low-frequency characteristics of the signal. Its fluctuation has a certain degree of smoothness, but there is still obvious sawtooth-like noise overall. Figure 18b shows the noise reduction results by EMD (black lines represent the denoised signal). The noise-reduced signal is extremely smooth. The noise in the original pressure signal has been significantly suppressed, but excessive smoothness can lead to signal distortion. The denoised signal deviates from the original pressure signal and loses the characteristics of the original signal. Figure 18c shows the denoising effect of the proposed method in this paper (black lines represent the denoised signal). From the figure, it can be seen that the denoising method proposed in this paper utilizes multi-level signal processing, combined with VMD decomposition, wavelet thresholding, and the black-winged kite optimization algorithm to quantify the complexity of the signal through sample entropy, ensuring accurate preservation of signal features. While effectively suppressing signal noise, it filters out sudden interference in the original pressure signal. Therefore, we believe that this method has significant advantages in handling complex and nonlinear signals.

## 4. Conclusions

In order to overcome the shortcomings of variational mode decomposition, which relies solely on empirical values to obtain key parameters during signal decomposition, resulting in unclear signal decomposition and unsatisfactory noise reduction effect, this paper proposes a novel VMD parameter combination optimization method based on the black-winged kite algorithm, taking the pressure signal of the water supply pipeline as the research object. This method introduces a fitness function based on minimum envelope entropy to optimize the selection of VMD parameters and improve the decomposition accuracy of the signal. Considering that, after denoising, there may be some noise present in the high-frequency range of the signal, in order to improve feature signal extraction, this paper uses wavelet thresholding for secondary denoising.

By constructing simulation signals and building experimental platforms to obtain leakage signals from water supply pipelines, the effectiveness of the proposed method for noise reduction is verified. In the process of noise reduction analysis of simulated signals, the experimental results show that the method proposed in the paper can achieve the highest SNR of 14.2280 dB, compared to WT’s SNR of 12.6458 dB and EMD’s SNR of 5.5292 dB. In addition, in the pipeline leakage signals collected in the laboratory, wavelet denoising, EMD, and the method proposed in this paper were used to denoise the pressure signals in the leakage state. The experimental results show that the EMD method can cause significant distortion of the signal after denoising, and the effect of wavelet denoising does not meet expectations. In contrast, the method proposed in this paper performs the best in denoising, effectively preprocessing the features of pressure signals while smoothing the denoised signals, providing a reliable basis for subsequent leak identification and localization. The research in this paper has strong practical significance.

Currently, our research is only conducted in the laboratory and has not yet covered issues such as computational complexity, real-time feasibility, and robustness against sensor failures or long-term drift. In order to better apply our method to the monitoring of real pipeline operation conditions and enhance the practicality of this method, we will conduct research on these aspects in the next step of our work.

## Figures and Tables

**Table 1:** Optimization results of different optimization algorithms.

	IBWK-VMD		BWK-VMD		NGO-VMD	
	α	*K*	α	*K*	α	*K*
	3455	5	2785	7	2293	9
	4132	6	4006	8	3253	7
	3155	4	5000	4	5000	4
	2761	4	2184	6	1709	5
	2138	5	3199	9	2327	7
	3041	6	4412	9	4409	7
Mean	3113.67	5.00	3317.20	7.16	3165.17	6.50
Standard deviation	668.85	0.89	901.99	1.94	1304.23	1.76

**Table 2:** SNR of noise reduction signal by different methods.

SNR of Simulated Signals (dB)	Methods	SNR After Noise Reduction (dB)	SNR Improvement (dB)
4	WT	12.6458	8.6458
	EMD	5.5292	1.5292
	IBWK-VMD-WT	14.2280	10.2280

**Table 3:** The entropy of each IMF sample.

IMF	SpEn
IMF1	0.4985
IMF2	0.6427
IMF3	0.6507
IMF4	0.6687
IMF5	0.6576
IMF6	0.0026

## Data Availability

Dataset available on request from the authors.
